# Expression of thymidylate synthase and orotate phosphoribosyltransferase in thymic carcinoma

**DOI:** 10.3892/etm.2012.641

**Published:** 2012-07-19

**Authors:** KEISUKE YOKOTA, HIDEFUMI SASAKI, KATSUHIRO OKUDA, MASAYUKI SHITARA, YU HIKOSAKA, SATORU MORIYAMA, MOTOKI YANO, YOSHITAKA FUJII

**Affiliations:** Department of Oncology, Immunology and Surgery, Nagoya City University Graduate School of Medical Sciences, Nagoya, Japan

**Keywords:** thymic carcinoma, thymidylate synthase, orotate phosphoribosyltransferase

## Abstract

Thymic carcinoma is a rare thymic epithelial tumor in which chemotherapy for advanced disease has not yet been established. Thymidylate synthase (TS) and orotate phosphoribosyltransferase (OPRT) protein expression levels in thymic carcinoma were evaluated as possible indicators of the anticancer activity of 5-fluorouracil (5-FU) drugs using immunohistochemistry (IHC). A total of 24 samples of thymic carcinoma were used in the present study. The tumor sections were immunohistochemically stained for TS and OPRT. As a comparison with thymic carcinoma, we also assessed the TS and OPRT protein expression levels in 55 lung cancer samples. The TS expression was positive in 12 of 24 thymic carcinoma samples (50%) and OPRT expression was positive in 10 (42%). The association between TS and OPRT expression and Masaoka stages of thymic carcinoma was analyzed. The TS and OPRT expressions in stage IV were significantly higher compared to that in stages I, II or III. We also compared the TS and OPRT expression levels between thymic carcinoma and lung cancer (33 adenocarcinomas and 22 squamous cell carcinomas). TS expression in thymic carcinoma was significantly lower compared with lung squamous cell carcinoma. OPRT expression in thymic carcinoma was significantly higher compared to lung adenocarcinoma. The combination of a relatively low expression of TS and high expression of OPRT suggests an improved antitumor effect of 5-FU drugs in thymic carcinoma compared to in lung carcinoma.

## Introduction

Thymic carcinoma is a rare epithelial tumor of the thymus. Its incidence appears to be higher in Asians than in Caucasians (1). Completeness of resection has been considered to be the most important determinant of long-term survival in thymic carcinoma (2,3). Systemic chemotherapy or radiation therapy is usually selected for the patients with an unresectable or metastatic disease. However, an optimal chemotherapeutic drug or regimen has not yet been determined for advanced or recurrent thymic carcinoma. Previous studies of chemotherapy for advanced thymic carcinoma were based on the regimens for advanced thymoma or germ cell tumor, using cisplatin-based chemotherapy (4–6). Reports of secondary or salvage chemotherapy have been single case reports or for thymic carcinomas of a small size (7–11).

Recently, Okuma *et al* (12) and Koizumi *et a*l (13) reported the usefulness of oral S-1 monotherapy as second-line or later chemotherapy for advanced thymic carcinoma. S-1 is an oral fluoropyrimidine agent composed of tegafur, 5-chloro-2,4-dihydroxypyridine (CDHP) and potassium oxonate. Tegafur, which is a prodrug of 5-fluorouracil (5-FU), is converted to 5-FU *in vivo*. One of the targets of 5-FU is thymidylate synthase (TS). Tegafur is rapidly catabolized by dihydropyrimidine dehydrogenase (DPD). CDHP, a component of S-1, inhibits DPD and, thus, maintains high 5-FU activity. 5-FU activity is accelerated by orotate phosphoribosyltransferase (OPRT) which converts 5-FU to its active form. Thus, the anticancer activity of S-1 is influenced by the expression levels of TS and OPRT.

In order to estimate the anticancer activity of 5-FU drugs against thymic carcinoma, TS and OPRT protein expression levels were investigated in thymic carcinomas using immunohistochemistry (IHC).

## Materials and methods

### Patients and clinicopathological characteristics

Thymic carcinoma tissue samples from 24 patients who underwent surgery or core-needle biopsy between 1986 and 2009 at Nagoya City University Hospital (Nagoya, Japan) were used in the present study. All patients consented to the use of their tissues for the analysis. The patients consisted of 12 males and 12 females with a median age of 60 years, ranging from 33 to 84. Pathological diagnosis revealed squamous cell carcinoma in 13, adenocarcinoma in 2, and other types of carcinoma (mucoepidermoid, neuroendocrine and lympho epithelioma-like carcinomas) in 3 patients. Detailed pathological findings were not determined for 6 patients. The patients were staged according to the Masaoka clinical staging system with the following results: 1 patient in stage I, 5 in stage II, 8 in stage III, 2 in stage IVa and 8 in stage IVb. With regard to treatment, 15 patients underwent surgery. For post-surgical treatment, 3 of 15 patients had adjuvant radiotherapy, 3 had chemotherapy and 1 had chemoradiotherapy. As induction therapy prior to surgery, 4 patients underwent chemotherapy and radiation therapy and 1 had chemotherapy. Nine patients were regarded as inoperable and 6 of these 9 patients underwent chemoradiotherapy, 2 had radiation therapy and 1 had chemotherapy. Among the 24 patients, no patient received chemotherapy with 5-FU drugs.

To study the difference between thymic carcinoma and carcinomas of other organs, 55 samples of lung carcinoma were compared with the samples of thymic carcinoma. These 55 patients included 33 adenocarcinomas and 22 squamous cell carcinomas. The 33 adenocarcinoma patients all underwent surgery and chemotherapy between 2003 and 2009. The 22 squamous cell carcinoma patients underwent surgery between 2003 and 2009. The clinicopathological characteristics of the 55 lung carcinoma patients were as follows: i) there were 33 males and 22 females, ii) pathological stage was diagnosed as I, II and III in 34, 15 and 6 patients, respectively and iii) the regimen of adjuvant chemotherapy was UFT in 33 patients, TS-1 in 5 patients and CBDCA+PTX in 4 patients.

### TS immunohistochemistry

TS protein expression was evaluated by IHC using recombinant human TS-specific antibody (clone RTSSA; dilution, 1:1,500; Taiho Pharmaceutical, Co., Ltd., Saitama, Japan). A standard protocol was used for immunostaining the 4-μm-thick paraffin-embedded tissue sections of thymic carcinoma. The sections were deparaffinized in xylene, dehydrated in ethanol, heated in a microwave for antigen retrieval using pH 6.0 citrate buffer solution, incubated with 0.3% hydrogen peroxidase in methanol to block endogenous peroxidase activity and incubated with the blocking solution (10% Block Ace) to block nonspecific binding. RTSSA was applied as the primary antibody and the slides were incubated overnight at 4°C. The slides were incubated with EnVision™ as the second antibody for 45 min at room temperature and visualized with 3,3′-diaminobenzidine and counterstained with hematoxylin.

The slides were examined at low magnification and the intensity of cytoplasmic staining was scored as follows: 0, no staining or faint staining; 1+, moderate staining; 2+, strong staining. We classified scores of 0 as negative and scores of 1+ and 2+ as positive for the TS antibody. We also evaluated cases with <10% of tumor cells with moderate or strong staining as negative.

### OPRT immunohistochemisrty

OPRT protein expression was evaluated by IHC using anti-OPRT polyclonal antibody (dilution, 1:1,200; Taiho Pharmaceutical, Co., Ltd.). The staining procedure was the same as for TS, with the exception of the primary antibody.

The scores used for the intensity of cytoplasmic staining were the same as for TS. Scores of 0 and 1+ were classified as negative and scores of 2+ as positive for the OPRT protein. We also evaluated cases with <10% of tumor cells with moderate or strong staining as negative.

### Statistical analysis

Survival curves were generated using the Kaplan-Meier method and the log-rank test was used to determine statistical significance of the difference between groups. A log-rank test was used to compare the survival distributions of the two groups. The Mann-Whitney U test was used to assess whether there was a significant difference in the median values between the two independent samples. The two-sided significance level was at P<0.05. All of the analyses were performed using Ekuseru-Toukei 2010 of Excel software.

## Results

### Immunohistochemical analysis of TS expression of thymic carcinoma

**Figure 1:**
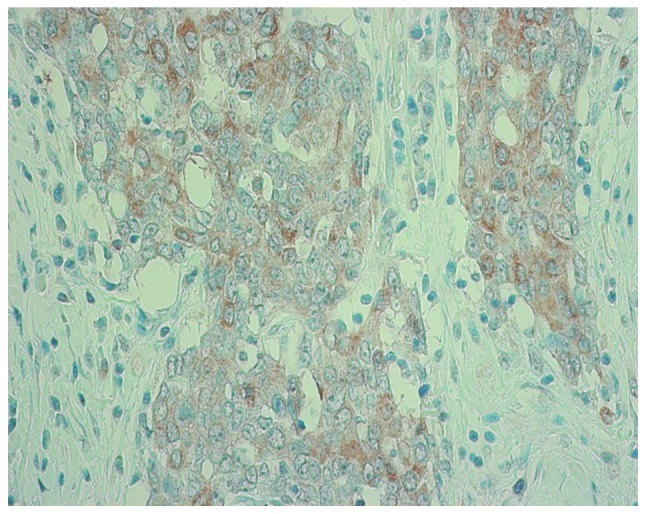
Immunohistochemical staining of TS in thymic carcinoma (IHC score, 2+). TS, thymidylate synthase; IHC, immunohistochemistry.

**Figure 2:**
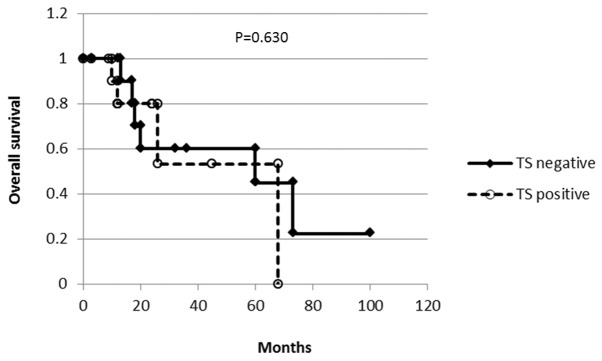
Kaplan-Meier estimates of overall survival according to TS expression. TS, thymidylate synthase.

The immunostaining scores were as follows: 12 samples were scored as 0, 10 as 1+ and 2 as 2+. Those with a score of 1+ or 2+ were considered TS-positive. In all of these samples, staining was counted over 10% of tumor cells. Fig. 1 shows a representative staining of one sample which was scored as 2+. The association between TS expression and overall survival was analyzed with the Kaplan-Meier method (Fig. 2). No difference was observed in survival according to the TS staining (P=0.630).

The association between TS protein expression and Masaoka stages (stage I, II and III vs. stage IV) was analyzed for the difference in TS expression between the stages. The tumors of stage IV showed significantly higher TS expression than stages I, II and III (Table I).

### Immunohistochemical analysis of OPRT expression of thymic carcinoma

**Figure 3:**
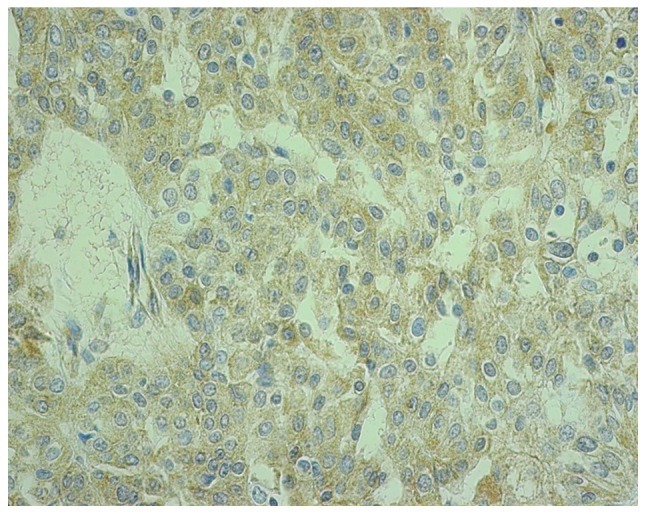
Immunohistochemical staining of OPRT in thymic carcinoma (IHC score, 2+). OPRT, orotate phosphoribosyltrasferase; IHC, immunohistochemistry.

**Figure 4:**
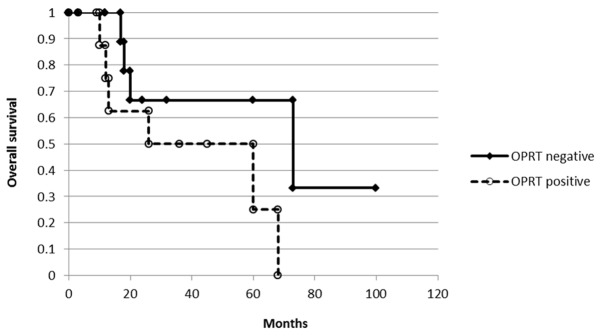
Kaplan-Meier estimates of overall survival according to OPRT expression. OPRT, orotate phosphoribosyltransferase.

The immunostaining scores were as follows: 4 samples were scored as 0, 10 as 1+ and 10 as 2+. Samples with a score of 0 or 1+ were considered as OPRT-negative and those with 2+ as OPRT-positive. In all of these samples, staining was counted over 10% of tumor cells. Fig. 3 shows a representative staining of score 2+. The association between OPRT expression and overall survival was analyzed with the Kaplan-Meier method (Fig. 4). There was no difference according to the OPRT staining (P= 0.101) but there was a tendency for OPRT-negative cases to demonstrated a longer survival than OPRT-positive cases.

The association between the OPRT protein expression and Masaoka stage was analyzed. The tumors at stage IV showed a significantly higher OPRT expression than those at stage I, II or III (Table II).

### Comparison of TS expression between thymic carcinoma and non-small cell lung cancer (NSCLC)

**Figure 5:**
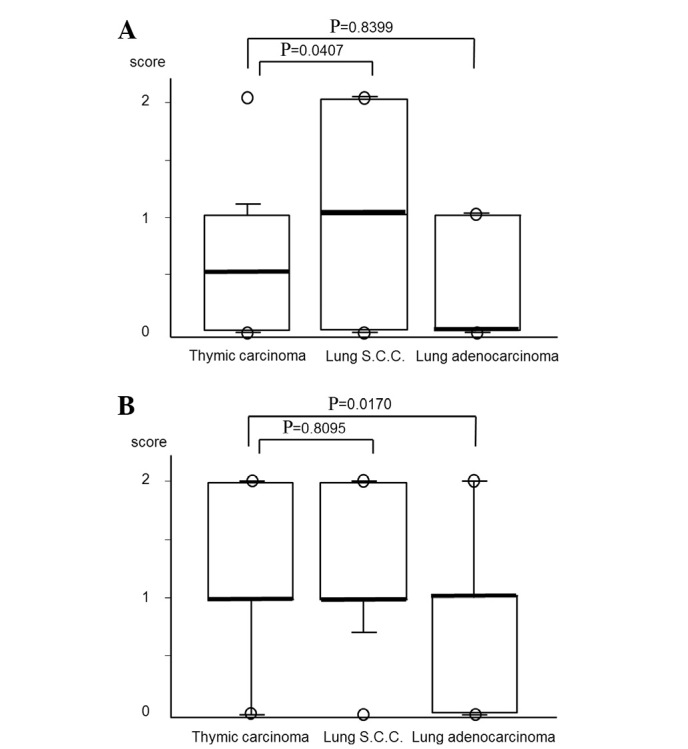
(A) Comparison of mean scores of TS between thymic carcinoma and lung cancer. Mean score of TS in lung squamous cell carcinoma was significantly higher than that in thymic carcinoma. (B) Comparison of mean scores of OPRT between thymic carcinoma and lung cancer. Mean score of OPRT in thymic carcinoma was significantly higher than that in lung adenocarcinoma. S.C.C., squamous cell carcinoma; TS, thymidylate synthase; OPRT, orotate phosphoribosyltransferase.

To examine the differences in TS expression between thymic carcinoma and NSCLC, TS protein expression was analyzed for the 33 lung adenocarcinomas and 22 lung squamous cell carcinomas. The TS expression scores of the 33 lung adenocarcinomas were as follows: 16 samples were scored as 0 and 17 as 1+. With regard to the 22 lung squamous cell carcinomas, 7 samples were 0, 6 as 1+ and 9 as 2+. The TS protein expression of lung squamous cell carcinoma was significantly higher than that in thymic carcinoma (P= 0.0407), whereas there was no difference between thymic carcinoma and lung adenocarcinoma (Fig. 5A).

The differences in TS expression between thymic squamous cell carcinoma and NSCLC were also assessed. TS protein expression of lung squamous cell carcinoma was significantly higher than that in thymic squamous cell carcinoma (P=0.0358), whereas there was no difference between the thymic squamous cell carcinoma and lung adenocarcinoma (data not shown).

### Comparison of OPRT expression between thymic carcinoma and NSCLC

To examine the differences of OPRT expression between thymic carcinoma and NSCLC, OPRT protein expression of the 33 lung adenocarcinoma and 22 lung squamous cell carcinomas was analyzed. OPRT expression in the lung adenocarcinomas was as follows: 11 samples were scored as 0, 18 as 1+ and 4 as 2+. With regard to the lung squamous cell carcinoma samples, 2 were scored as 0, 11 as 1+ and 9 as 2+. OPRT protein expression in thymic carcinoma was significantly higher than that in lung adenocarcinoma (P=0,0170), whereas there was no difference between the thymic carcinoma and the lung squamous cell carcinoma (Fig. 5B).

The differences in OPRT expression between thymic squamous cell carcinoma and NSCLC were also assessed. There was no difference between the thymic squamous cell carcinoma and NSCLC, lung squamous cell carcinoma or lung adenocarcinoma (data not shown).

## Discussion

The TS and OPRT protein expression of the 24 thymic carcinomas was evaluated with the aim of predicting the effect of 5-FU drugs for thymic carcinomas.

As thymic carcinoma is a rare thymic epithelial tumor, a clinical trial of chemotherapy for advanced or recurrent cases is difficult to plan. In addition, no clinical trial on a large scale has been conducted due to the difficulties in recruiting patients. There have been case reports of effective S-1 treatments for thymic carcinoma. S-1 has been used for patients with advanced lung cancer (14). In lung cancer, TS and OPRT expression has been reported to be related to the effect of 5-FU drugs (15–17). In thymic carcinoma, however, TS and OPRT expression has not yet been reported.

TS is the enzyme that generates deoxythymidine monophosphate (dTMP), which is subsequently phosphorylated to thymidine triphosphate for use in DNA synthesis and repair. High levels of TS expression have been reported in association with aggressiveness (18), metastasis and poor prognosis in various cancers (15–17,19,20). A tumor with high TS expression tends to be resistant to a TS inhibitor compared with a tumor showing low TS expression. TS expression in squamous cell carcinoma has been shown to be higher than in adenocarcinoma in general (21,22), suggesting its relative resistance to 5-FU.

In the present study, TS and OPRT expression was demonstrated in 24 thymic carcinomas using immunohistochemistry. The TS expression in advanced-stage thymic carcinoma was significantly higher than those in early stages, suggesting resistance to 5-FU. Similar results have been reported with other types of cancer (15–17,19). We compared TS expression of thymic carcinoma with lung adenocarcinoma and squamous cell carcinoma. The level of TS expression in thymic carcinoma was similar to lung adenocarcinoma but significantly lower than in squamous cell carcinoma. These results indicate the possibility of using S-1 as a therapeutic drug for thymic carcinoma. These data may be relevant to the antitumor effect of another anticancer drug, pemetrexed, which is also approved for NSCLC. Pemetrexed is chemically similar to folic acid and is classified as a folate antimetabolite. Pemetrexed inhibits three enzymes including TS which are used in purine and pyrimidine synthesis (23). It is approved for lung adenocarcinoma, with a lower level of TS expression, but is not approved for lung squamous cell carcinoma having a higher level of TS expression.

OPRT is an enzyme involved in pyrimidine biosynthesis and contributes to the conversion of 5-FU into fdUMP, an active form of 5-FU. In the present study, OPRT expression of thymic carcinoma was significantly higher in patients of advanced stage than in those of earlier stages. OPRT expression in thymic carcinoma was significantly higher than in lung adenocarcinoma, and was similar to that of squamous cell carcinoma. Thymic carcinoma was shown to have a combination of relatively low expression of TS and high expression of OPRT and is likely to be sensitive to antitumor drugs such as 5-FU.

TS expression in thymic carcinoma was higher in more advanced stages. This appears to be useful in predicting that thymic carcinomas at an advanced stage are more resistant to S-1 than those in early stages. However, as OPRT expression was also higher in more advanced stages, 5-FU is more highly activated and subsequently may be effective in advanced stages.

While the results of this study are encouraging, it is acknowledged that any conclusions should be tempered with certain reservations. The small number of samples limited the statistical power of the study. Thymic carcinoma is a rare disease and it is difficult to undertake a large scale study. Our study was confined to the protein expression of the enzymes TS and OPRT using immunohistochemical analysis. Since these may be regulated at various levels, a further study to examine the expression of mRNA, by using *in situ* hybridization (ISH), may enhance the results of the present study.

In conclusion, we evaluated TS and OPRT protein expression in thymic carcinoma. The combination of relatively low expression of TS and high expression of OPRT suggests a sensitivity of thymic carcinoma to 5-FU drugs.

## Figures and Tables

**Table I:** Association between TS protein expression and Masaoka stage.

	Masaoka stage	
TS score	I, II, III	IV
0	10/14	2/10
1	4/14	6/10
2	0/14	2/10
Median score	0	1

P=0.0089. TS, thymidylate synthase.

**Table II:** Association between OPRT protein expression and Masaoka stage.

	Masaoka stage	
OPRT score	I, II, III	IV
0	4/14	0/10
1	7/14	3/10
2	3/14	7/10
Median score	1	2

P=0.0111. OPRT, orotate phosphoribosyltransferase.
